# The healthfulness of major food brands according to Health Canada’s nutrient profile model for proposed restrictions on food marketing to children

**DOI:** 10.1017/S1368980024002659

**Published:** 2025-01-07

**Authors:** Laura Vergeer, Christine Mulligan, Hayun Jeong, Ayesha Khan, Mary R L’Abbé

**Affiliations:** Department of Nutritional Sciences, Temerty Faculty of Medicine, University of Toronto, Toronto, ON, Canada

**Keywords:** Food marketing, Brand marketing, Marketing to children, Nutrient profile model, Food marketing policy, Canada

## Abstract

**Objective:**

To examine the proportion of products offered by leading food brands in Canada that are ‘unhealthy’ according to Health Canada’s (HC) nutrient profile model for proposed restrictions on food marketing to children (M2K-NPM).

**Design:**

Nutritional information for products offered by top brands was sourced from the University of Toronto FLIP and Menu-FLIP 2020 databases, respectively. HC’s M2K-NPM, which includes thresholds for Na, total sugars and saturated fat, was applied to products.

**Setting:**

Canada.

**Participants:**

Overall, 1385 products from top breakfast cereal (*n* 15 brands, *n* 222 products), beverage (*n* 21 brands, *n* 769 products) and yogurt (*n* 10 brands, *n* 394 products) brands, and 3153 menu items from seventeen chain restaurants in Canada were assessed (*n* 60 unique brands overall).

**Results:**

For 42 % of brands (*n* 21), 100 % of their products exceeded ≥1 nutrient threshold(s), with ≥50 % of the products offered by twenty-three brands (46 %) exceeding two thresholds. Specifically, one or more nutrient thresholds were exceeded by ≥50 % of the products offered by 14/15 breakfast cereal brands, 18/21 beverage brands, all ten yogurt brands and all seventeen restaurant brands. Notably, 100·0 % of the products offered by ten breakfast cereal, six beverage, two yogurt and three restaurant brands exceeded ≥1 threshold(s).

**Conclusions:**

Most products offered by top food brands in Canada exceeded HC’s M2K-NPM thresholds. Nonetheless, these brands could still be marketed under the proposed regulations, which exclude brand marketing (i.e. promotions without an identifiable product) despite its contribution to marketing power. These findings reinforce the need for Canada and other countries to include brand marketing in M2K policies.

An unhealthy diet is a major risk factor for obesity and other non-communicable diseases in Canada, including in children^(1)^. The diet quality of Canadian children is typically characterised by high intakes of ultra-processed foods that are energy-dense and high in Na, saturated fat and/or free sugars, as well as by inadequate intakes of fruits and vegetables^(2,3)^. One contributor to poor diet quality in children is the powerful marketing of unhealthy foods to which they are frequently exposed^(4)^. Marketing of unhealthy foods to children contributes to increased purchases (or purchase requests) for foods of lower nutritional quality, as well as less healthy food preferences, dietary choices and consumption patterns, ultimately increasing children’s risk of developing obesity and other non-communicable diseases^(5)^.

Accordingly, marketing of unhealthy foods to children has been identified as a critical public health concern and an increasing number of countries are implementing restrictions to limit children’s exposure to unhealthy food marketing^(5–7)^. The WHO recently released a new guideline on developing and implementing policies to protect children from marketing of unhealthy foods and recommended that countries introduce mandatory policies in this area^(5)^. In Canada, mandatory restrictions on food marketing to children only exist in Quebec, under the province’s Consumer Protection Act, which bans advertising of all goods and services (including food) directed at children under 13 years of age^(8)^. The rest of the country is limited to industry self-regulation of food marketing to children. In 2007, the Canadian Children’s Food and Beverage Advertising Initiative (CAI) was introduced as a voluntary commitment among sixteen participating food, beverage and restaurant companies to only advertise products that meet their nutrition criteria to children under 13 years old^(9)^. A new industry-wide voluntary self-regulatory code, the Code for the Responsible Advertising of Food and Beverage Products to Children (CCFBA), was launched by Canadian food companies in June 2023 to replace the CAI^(10)^. As part of the CCFBA, food advertisers can only promote products to children under 13 years of age that meet the code’s nutrition criteria. These self-regulatory codes are based on nutrition criteria developed by participating companies and administered by Ad Standards, a Canadian not-for-profit advertising self-regulatory organisation. However, several studies in Canada and elsewhere have demonstrated the ineffectiveness of the CAI and other voluntary self-regulatory commitments in limiting children’s exposure to unhealthy food marketing^(11)^.

The Canadian federal government first proposed mandatory restrictions on food marketing to children in 2016 through its Healthy Eating Strategy Initiative; however, these regulatory changes have yet to be approved^(7,12)^. The most recent proposed policy in this area, Bill C-252 (introduced in February 2022), is currently proceeding through Parliament^(12)^. If passed, Bill C-252 would prevent foods exceeding certain thresholds for Na, added fat and/or total sugars (if they contain added Na, added fat and/or free sugars) from being promoted in television or digital media advertisements primarily directed at children under the age of 13 years. Notably, advertisements that only promote a food brand (e.g. using a logo, slogan, spokes character or sponsorship) and do not visually or verbally identify or reference a particular product by name (e.g. by showing product packaging) would not be subject to the restrictions^(7)^. Brand marketing is also excluded from Quebec’s Consumer Protection Act and voluntary food marketing policies in Canada.

Excluding brand marketing from Health Canada’s (HC) proposed regulations may significantly lessen the potential for this policy to protect Canadian children from unhealthy food marketing. There is a growing body of literature to suggest that branding contributes to the power (i.e. the persuasiveness or effectiveness) of a marketing communication, message or action^(5,6,13,14)^. The WHO recently expanded its previous definition of marketing such that it now extends to not just the promotion of a product or service but also its related brand^(5)^. Food companies employ various marketing strategies to foster brand recognition and appeal, even in children^(6)^. As a result of successful brand advertising, many food and beverage products have become inextricably linked to a particular brand name and logo, establishing brand preference and loyalty among consumers, beginning as early as childhood^(15,16)^. Studies have indicated that children are knowledgeable about food brands and can identify food and beverage products by brand name^(5)^. The growth of digital food advertising in recent years is perpetuating this problem, by encouraging users (including children) to interact with a brand and promote it within their social networks^(13,17)^. Concerningly, brand advertising influences children’s recognition of and preference for these products and can have detrimental impacts on their food purchasing behaviours and consumption^(18)^. There is therefore increasing recognition of the importance of including brand marketing in policies aimed at restricting unhealthy food marketing to children, such that these policies apply to all instances of unhealthy food marketing with brand elements (e.g. logos, slogans, spokes characters and sponsorship)to which children are exposed, irrespective of whether an identifiable product is featured, as recommended by the WHO^(5,6)^. Brand marketing restrictions may, for example, prohibit unhealthy food brand logos from appearing on sports jerseys (e.g. Tim Hortons’ Timbits Sports), or ban television or digital media advertisements for unhealthy food brands featuring brand elements (e.g. the McDonalds famous golden arches logo, Kellogg’s Tony the Tiger), even if a specific food product is not shown or referenced. Excluding branding from food marketing regulations may not only leave children vulnerable but could also have detrimental consequences, such as more frequent and powerful brand marketing and sponsorship by brands known for less healthy products (e.g. soft drink brands or fast-food chains) to counteract food marketing restrictions^(5)^. The need for regulations that include brand marketing is reinforced by the fact that many existing self-regulatory codes generally do not extend to brand marketing, are limited by low age thresholds (despite the WHO recommending policies protect youth up to 18 years of age) and do not encompass media such as product packaging, social media influencer marketing or sponsorship of sporting/cultural events where children are present^(19)^.

The purpose of this study was to examine the healthfulness of products offered by leading packaged food, beverage and restaurant brands in Canada using HC’s nutrient profile model for restricting food marketing to children. Specifically, this work aimed to examine the proportion of products under each brand that would be considered too unhealthy to be marketed to children under the proposed regulations, with the ultimate goal of providing timely evidence to help inform policymakers.

## Methods

### Food composition data

This study was a cross-sectional analysis of data from the University of Toronto Food Label Information and Price (FLIP) 2020 and the Menu-FLIP 2020 databases, described in detail elsewhere^(20,21)^. In summary, FLIP 2020 is a branded food composition database that was developed using website scraping (‘web-scraping’) to collect information on the food labels of all food and beverage products available on seven major Canadian e-grocery retailer websites (representing >80 % of the grocery retail market share) between May 2020 and February 2021 (*n* 74 445 product listings; *n* 48 829 unique products). FLIP contains information on the product name, Universal Product Code, brand, nutritional composition (based on the Nutrition Facts table (NFt)), ingredients list, container size, price (regular and sale price) and images of the product packaging (as available). Information available in photo format only (e.g. NFt and ingredients lists from some websites) was extracted using Artificial Intelligence (AI)-enhanced/powered Optical Character Recognition (OCR) (AI-enhanced OCR) technology. All available food and beverage products were collected, including both national and private label brands, multiple package sizes and all flavours and varieties of a product. Nutrition information in FLIP is recorded ‘as sold’ (i.e. as indicated on the NFt at the time of purchase) and ‘as prepared’ (calculated based on preparation instructions listed on the product packaging). Products in FLIP were categorised according to the major (*n* 24) and minor (*n* 153) food categories in HC’s Table of Reference Amounts for Foods (TRA)^(22)^.

Menu-FLIP 2020 is a database for monitoring the nutritional composition of Canadian chain restaurant foods. Restaurants with ≥20 outlets as of 2020 (*n* 201) and with publicly available nutrition information online (*n* 141) were included, resulting in the collection of data on 18 760 menu items. Nutrition information was extracted from online menus by either an optical character recognition tool or manually by research assistants in November and December 2020. Identical menu items of the same size and items with missing or inaccurate nutrition information (identified using Atwater calculations) were excluded. Items in Menu-FLIP were categorised as one of the following: starters, entrees, sides, desserts and beverages.

### Brand data

Products manufactured by brands holding ≥1 % of the 2022 Canadian market share for breakfast cereals (*n* 15), soft drinks (including juices; *n* 21), yogurts (*n* 10) and chained consumer food service (*n* 17) were selected for analysis, based on data from Euromonitor International (Table 1). The brands in this sample represent a combined 71·4 % of the national market share for breakfast cereals, 50·3 % for soft drinks/juices (hereafter referred to as ‘beverages’), 85·7 % for yogurts and 62·1 % of the Canadian market share for consumer food service^(23–26)^. Breakfast cereals (TRA minor categories: C1, C2, C3 and C4), beverages (B1 and J11) and yogurts (D12 and D15) were selected because they have been shown in previous FLIP studies to contain a high proportion of products with child-appealing marketing^(27,28)^ and contain products of varying healthfulness (e.g. as opposed to categories such as confectionary or desserts, which are predominantly less healthy). Beverage brands that only sell plain bottled water were excluded (*n* 3).

**Table 1.** Brands included in the study sample with ≥1 % market share, including the company that owns each brand, as well as the brand’s Canadian market share and the number of products included in the analysis

Brand^*^	Company	Market share (%)*	Number of products (n)^†^
Breakfast cereals			
** Total**	**–**	**71·4**	**222**
Quaker	PepsiCo	11·9	50
Kellogg’s Special K	Kellogg	8·9	26
Cheerios	General Mills	8·1	32
Kellogg’s Corn Flakes	Kellogg	6·6	4
Harvest Crunch	PepsiCo	5·3	9
President’s Choice	Loblaw Companies	5·1	41
Post Shreddies	Post Foods	3·9	7
Kellogg’s Muslix	Kellogg	3·8	3
Kellogg’s Frosted Flakes	Kellogg	3·3	6
Kellogg’s Two Scoops Raisin Bran	Kellogg	3·3	4
Kellogg’s Froot Loops	Kellogg	3·2	6
Lucky Charms	General Mills	3·0	11
Compliments	Sobeys	1·9	13
Post Shredded Wheat	Post Foods	1·9	6
Post Honeycomb	Post Foods	1·2	4
Beverages			
** Total**	**–**	**50·3**	**769**
Tropicana	PepsiCo	6·3	35
President’s Choice	Loblaw Companies	4·4	129
Gatorade	PepsiCo	4·1	51
Oasis	A Lassonde	4·0	67
Nestea	Coca-Cola/Nestlé	3·6	22
Coca-Cola	Coca-Cola	3·1	24
Canada Dry	Canada Dry Mott’s	2·8	29
Pepsi	PepsiCo	2·6	19
Red Bull	Red Bull	2·5	12
Minute Maid	Coca-Cola	1·9	61
Perrier	Nestlé	1·9	38
Monster Energy	Coca-Cola	1·8	23
Diet Coke	Coca-Cola	1·5	27
Compliments	Sobeys	1·4	118
Ocean Spray	Ocean Spray Cranberries	1·4	45
Sun-Rype	A Lassonde	1·3	12
Diet Pepsi	PepsiCo	1·2	14
Pure Leaf	Unilever	1·2	16
Snapple	Keurig Dr Pepper	1·2	4
Powerade	Coca-Cola	1·1	4
Simply	Coca-Cola	1·0	19
Yogurts			
** Total**	**–**	**85·7**	**394**
Iögo	Lactalis	15·6	54
Activia	Danone	15·3	76
Danone	Danone	13·6	8
Yoplait	General Mills	12·5	55
Liberté	General Mills	10·8	89
Astro	Lactalis	8·6	42
DanActive	Danone	4·1	3
Olympic	Lactalis	1·8	21
Danone Go	Danone	1·7	2
President’s Choice	Loblaw Companies	1·7	44
Restaurants			
** Total**	**–**	**62·1**	**3153**
Tim Hortons	Restaurant Brands International	19·5	269
McDonald’s	McDonald’s	13·5	225
A&W	A&W Food Services of Canada	4·3	114
Subway	Doctor’s Associates	4·0	188
Starbucks	Starbucks	2·9	640
Boston Pizza	Boston Pizza International	2·6	55
KFC	Yum! Brands	1·7	100
Pizza Hut	Yum! Brands	1·7	486
Swiss Chalet	Recipe Unlimited	1·7	78
Wendy’s	Wendy’s	1·6	75
St Hubert	Recipe Unlimited	1·5	95
The Keg	Recipe Unlimited	1·4	111
Dairy Queen	International Dairy Queen	1·3	178
Domino’s Pizza	Domino’s Pizza	1·2	140
Burger King	Restaurant Brands International	1·1	161
Pizza Pizza	Pizza Pizza	1·1	163
Harvey’s	Recipe Unlimited	1·0	75

* Brands in the ‘breakfast cereals’, ‘soft drinks’ (includes juices), ‘yoghurt and sour milk products’ and ‘chained consumer foodservice’ categories with ≥1 % of the 2022 Canadian market share in these categories were selected for analysis^(23–26)^.

† The total number of products in the FLIP 2020 database (breakfast cereals, beverages and yogurts) or menu items in the Menu-FLIP 2020 database (restaurants) offered under each brand.

FLIP 2020 includes the brand name under which a product is offered. This information was captured either through web-scraping (e.g. when the brand name is listed on the product page of the grocery retailer website) or AI-enhanced OCR technology for photos (e.g. to discern a brand name from a photo of the product packaging). Brand data was subsequently validated by a research associate (LV) to ensure that the correct brand name was extracted. ‘Brand’ was defined according to the manufacturer’s Canadian website. For example, ‘Coca-Cola’ and ‘Diet Coke’ were considered two different brands since that is how they are listed on the Coca-Cola Canada website. They are also listed as separate brands in Euromonitor’s Canadian market share data^(24)^.

### Health Canada’s nutrient profile model for restricting marketing to children

The nutritional quality of products in this sample was assessed using HC’s nutrient profile model outlined in proposed restrictions on unhealthy food marketing to children^(7)^. As part of Bill C-252^(12)^, HC is proposing to prohibit the marketing of foods and beverages that exceed specified thresholds for Na, total sugars and/or saturated fat. These thresholds are equivalent to 6 % of the Daily Value (DV) for Na, 5 % of the DV for total sugars and 10 % of the DV for saturated fat. In accordance with the proposed regulations, each nutrient was evaluated independently and only products containing added Na, free sugars or fat were evaluated against the ‘low in’ nutrient content claim thresholds (hereinafter referred to as ‘HC’s M2K thresholds’ (HC’s marketing to kids thresholds)). Therefore, only products containing added Na (e.g. added Na or salt, monosodium glutamate, added cheese or salted nuts, baking soda) were assessed against the Na threshold, only products containing free sugars (e.g. 100 % and concentrated fruit juice, honey, sugars from artificial flavours or fruit and vegetable purées, dextrose) were assessed against the total sugars threshold and only those with added fat (e.g. vegetable and animal fats and oils, olive oil, butter, margarine, shortening) were assessed against the saturated fat thresholds. Foods containing no added Na, free sugars or added fat were therefore exempt from evaluation (e.g. whole or cut vegetables or fruit, whole grains, plain milk or yogurt, eggs; *n* 407 packaged food and restaurant menu items). Because items in Menu-FLIP typically lack ingredients lists, only products that could be reasonably assumed to contain no added Na, added fat or free sugars were exempted (e.g. raw apple slices and bottled water). Exempt products are considered permitted for marketing to children under HC’s proposed restrictions.

HC’s M2K thresholds are based on the reference amount for that product, as defined in HC’s TRA. A reference amount refers to a standard serving size for that minor food category, based on amounts typically consumed by Canadians in a single sitting^(22)^. As outlined in the draft regulations, foods with small reference amounts of 30 g or less (e.g. sauces, dips or condiments) were assessed based on 50 g. Foods with standard reference amounts (greater than 30 g, except for main dishes) were evaluated against either the reference amount or the serving size stated on the NFt or menu, whichever was greater. Foods classified as main dishes (i.e. restaurant meals, such as hamburgers, chicken tenders and pizza) with a reference amount ≥200 g were assessed at 100 g. The nutrient thresholds for all foods and beverages were as follows: ≤140 mg for Na, ≤5 g for total sugars and ≤2 g SFA and *trans*-fatty acids combined per reference amount or serving of stated size and <15 % energy from the sum of saturated and *trans*-fatty acids. For packaged foods and beverages, ‘as prepared’ nutrient values were used, where applicable (e.g. for beverage concentrates mixed with water). When restaurant meals contained multiple components (e.g. entrée, side), each was assessed against the nutrient thresholds individually (i.e. each component of the meal was considered a unique product).

### Study sample and analyses

**Figure 1.**
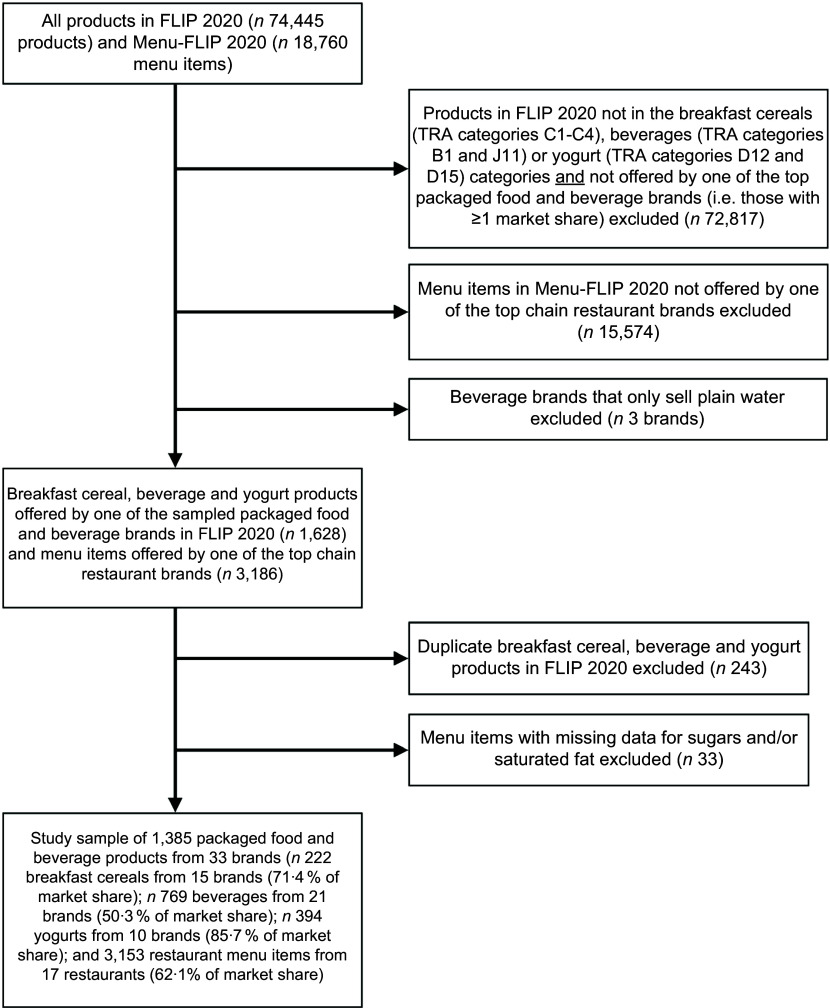
The approach used to derive the sample of brands and products examined in this study. TRA, Table of Reference Amounts for Foods; FLIP, Food Label Information and Price.

Products in FLIP offered by brands holding <1 % of the Canadian market share (for breakfast cereals, soft drinks or yogurts) or from food categories other than those of interest were excluded (*n* 72 817; Fig. 1). All package sizes, flavours and varieties of the sampled products in FLIP were included; however, when the exact same product was collected from multiple stores, it was analysed only once, resulting in the exclusion of 243 duplicate products. Similarly, menu items offered by restaurant brands other than those included in this sample were excluded from the analysis (*n* 15 574). Menu items with missing data for sugars (*n* 3 Pizza Hut items) and/or saturated fat (*n* 29 Pizza items; *n* 1 Starbucks item) were also excluded since information on these nutrients is required to apply HC’s nutrient profile model. The final analytic sample included 1385 packaged food and beverage products from forty-three brands (*n* 222 breakfast cereals from 15 brands; *n* 769 beverages from 21 brands; *n* 394 yogurts from 10 brands) and 3153 restaurant menu items from seventeen restaurants for a total of sixty unique brands (President’s Choice was among the top brands for breakfast cereals, beverages and yogurts, and Compliments was included in both the breakfast cereal and beverages brands) (Fig. 1).

All non-exempt products were evaluated against each of HC’s M2K thresholds for Na, saturated fat and total sugars. Products exceeding one or more nutrient thresholds were considered restricted from marketing to children, while exempt products and those that were not exempt but not in excess of Na, saturated fat or total sugars were considered permitted for marketing. The number and proportion of products under each brand that would be restricted and permitted for marketing to children under HC’s proposed nutrient thresholds were calculated. The number and proportion of each brand’s products that exceeded 0, 1, 2 or 3 nutrient thresholds and that exceeded the individual thresholds for Na, saturated fat and total sugars were also determined. A 50 % threshold was used in the reporting of results, such that the number of brands with 50 % or more products (in that food category) that would be restricted from marketing to children were considered to be brands comprised of mostly unhealthy foods in that category. IBM SPSS Statistics (version 29.0.1.0) was used to complete all analyses.

## Results

**Figure 2.**
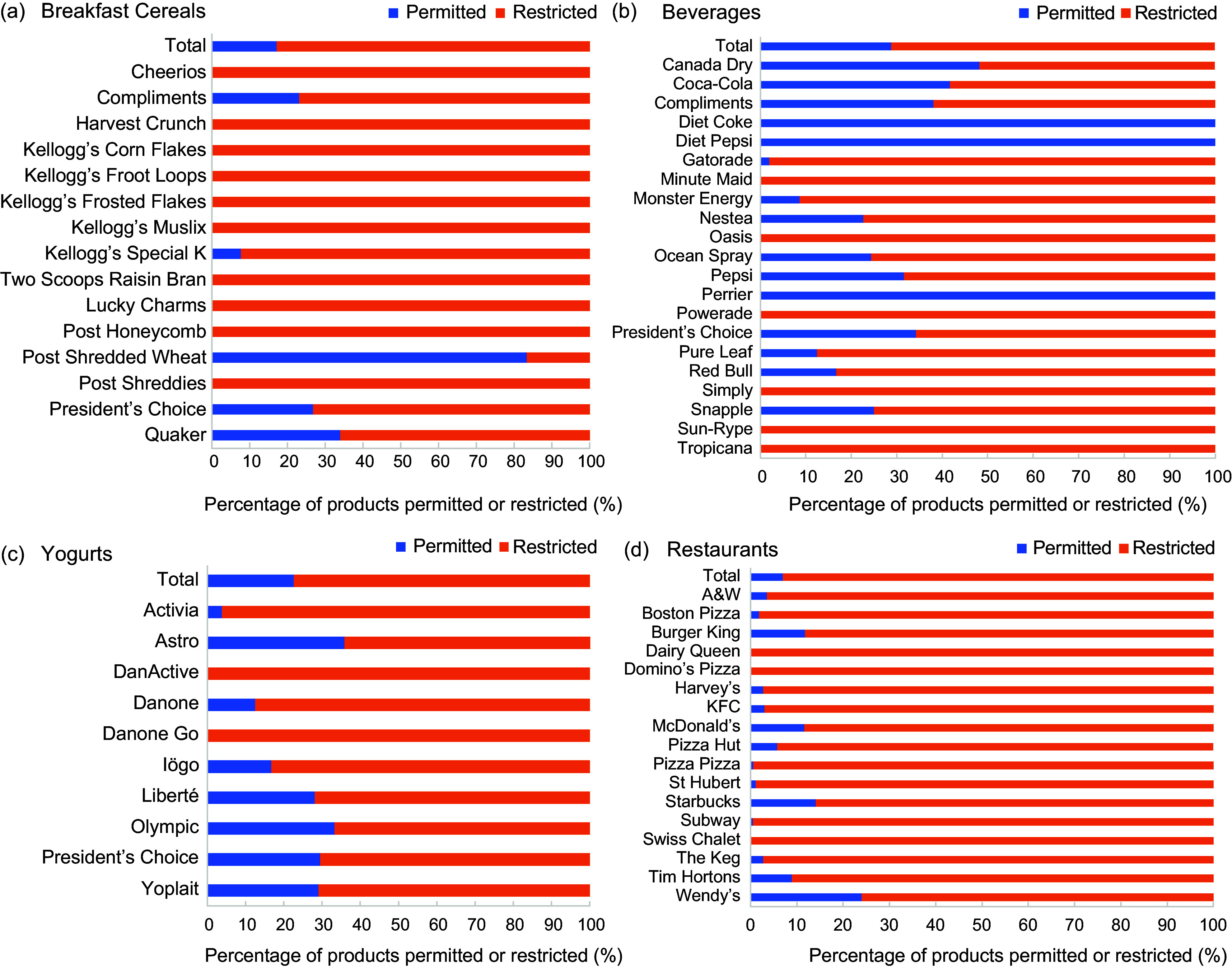
(a–d) The proportion of products offered the top breakfast cereal, beverage, yogurt and restaurant brands that would be permitted and restricted from marketing to children (M2K) based on the nutrient thresholds proposed by Health Canada (HC)*. ^*^Products or menu items without free sugars, added Na or added fat were exempted from all of HC’s M2K thresholds.

Overall, 74·8 % (*n* 1036) of the packaged foods and beverages in this sample would be restricted from marketing to children under the proposed regulations. Of the 25·2 % of products that would be permitted to be marketed to children, 16·3 % were exempt from evaluation (due to not containing added Na, free sugars or added fat, as per HC’s policy proposal)^(7)^ and 8·9 % were not exempt, but they did not exceed any of the nutrient thresholds (Fig. 2(a)–(d); see online supplementary material, Supplementary Table 1). Of the breakfast cereals included in this sample, 82·9 % would be restricted from being marketed to children. For fourteen of the fifteen breakfast cereal brands, 50 % or more of their products exceeded one or more nutrient thresholds, including 100·0 % of the products offered by ten brands. Among beverage products, 71·1 % would be restricted from being marketed to children (Fig. 2(a)–(d); see online supplementary material, Supplementary Table 1). For eighteen of the twenty-one beverage brands, more than 50 % of their products were found to be too unhealthy to be marketed, including 100·0 % of the beverages offered by six brands. Only diet soft drinks and carbonated water brands had no products that would be restricted from marketing to children (i.e. Diet Coke, Diet Pepsi and Perrier). Overall, 77·4 % of yogurt products would be restricted, including more than 50 % of the products offered by all ten yogurt brands and 100·0 % of the products offered by DanActive and DanoneGo (Fig. 2(a)–(d); see online supplementary material, Supplementary Table 1). Lastly, among the restaurants sampled, 93·0 % of their menu items would be restricted from marketing to children, with all seventeen restaurants having more than 50 % of their items considered too unhealthy to be marketed (Fig. 2(a)–(d); see online supplementary material, Supplementary Table 1). Additionally, 100·0 % of the menu items offered by Dairy Queen, Domino’s Pizza and Swiss Chalet exceeded one or more nutrient thresholds. Overall, all restaurant brands and all packaged food brands except for four brands (Post Shredded Wheat, Diet Coke, Diet Pepsi and Perrier) had 50 % or more of their products exceed at least one nutrient threshold.

**Figure 3.**
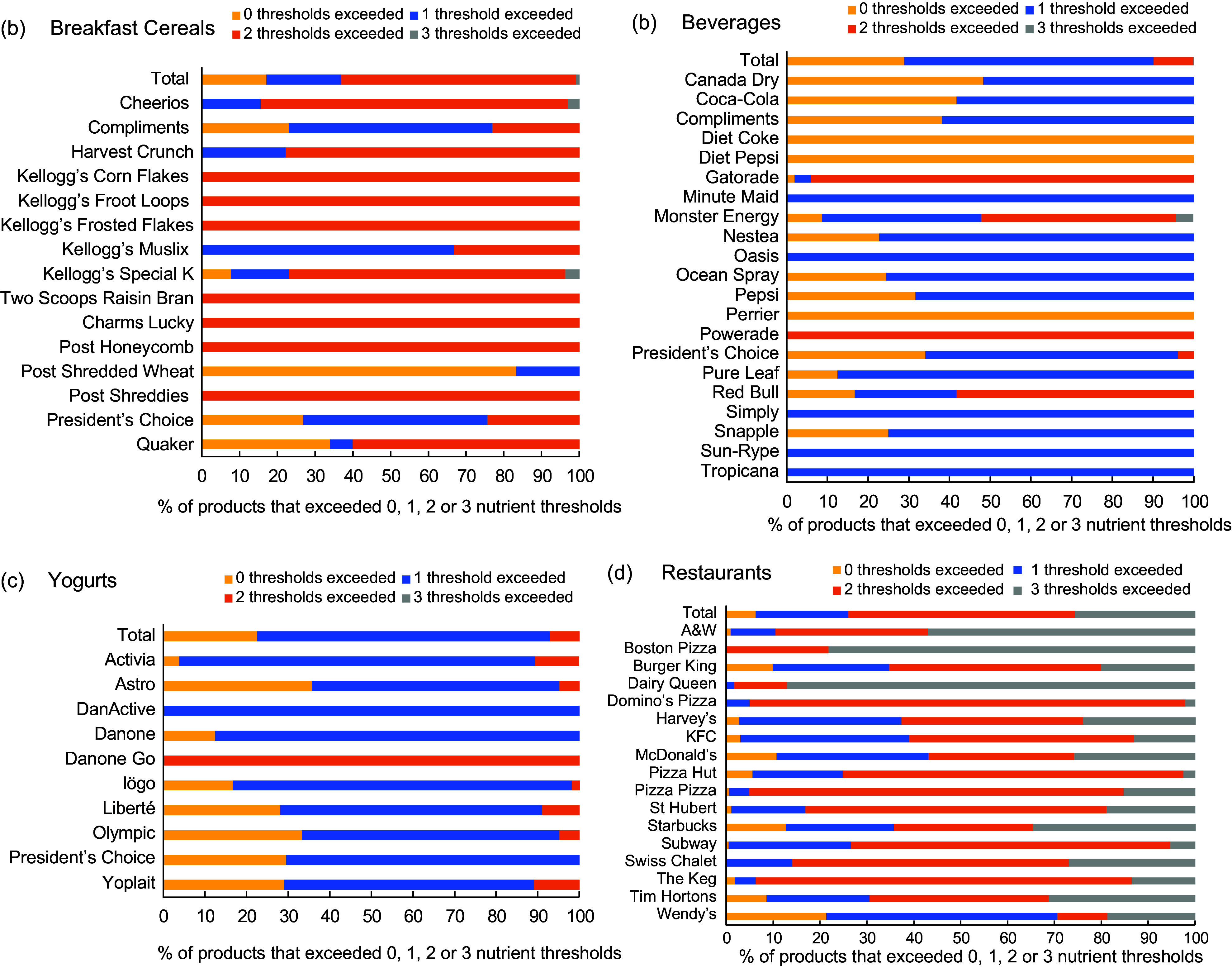
(a–d) The proportion of products offered by each breakfast cereal, beverage, yogurt and restaurant brand that exceeded 0, 1, 2 or 3 of Health Canada’s nutrient thresholds for proposed restrictions on food marketing to children. Products exceeding one or more nutrient thresholds would be restricted from marketing to children*. ^*^Products or menu items without free sugars, added Na or added fat were exempted from all of Health Canada’s nutrient thresholds. Products exceeding 0 thresholds include those that contained added Na, added fat and/or free sugars but did not exceed any nutrient thresholds, as well as products that did not contain added Na, added fat or free sugars and were therefore exempt from evaluation against the thresholds.

Among the total sample of packaged foods and beverages, 25·2 % of products exceeded no nutrient thresholds, 57·2 % exceeded one threshold, 17·4 % exceeded two thresholds and 0·2 % exceeded all three nutrient thresholds. For breakfast cereals, most products exceeded two nutrient thresholds (62·2 %), with 50 % or more of the products offered by eleven of the fifteen brands exceeding two thresholds (Fig. 3(a)–(d); see online supplementary material, Supplementary Table 2). Additionally, 100·0 % of the products offered by seven brands were found to exceed two thresholds. Most beverage products exceeded one nutrient threshold (61·2 %), including at least 50 % of the products offered by 15/18 brands and 100·0 % of the products offered by five brands (Fig. 3(a)–(d); see online supplementary material, Supplementary Table 2). Similarly, 70·3 % of yogurts exceeded one nutrient threshold, including 50 % or more of the products of all ten brands and 100·0 % of DanActive products (Fig. 3(a)–(d); see online supplementary material, Supplementary Table 2). Among restaurant menu items, 48·4 % exceeded two nutrient thresholds,and about one-quarter (25·6 %) exceeded three thresholds (Fig. 3(a)–(d); see online supplementary material, Supplementary Table 2). At least 50 % of the menu items offered by seven and three of the seventeen brands exceeded two and three nutrient thresholds, respectively. Notably, 87·5 % of Dairy Queen menu items exceeded all three nutrient thresholds, as did 78·2 % of Boston Pizza menu items.

**Figure 4.**
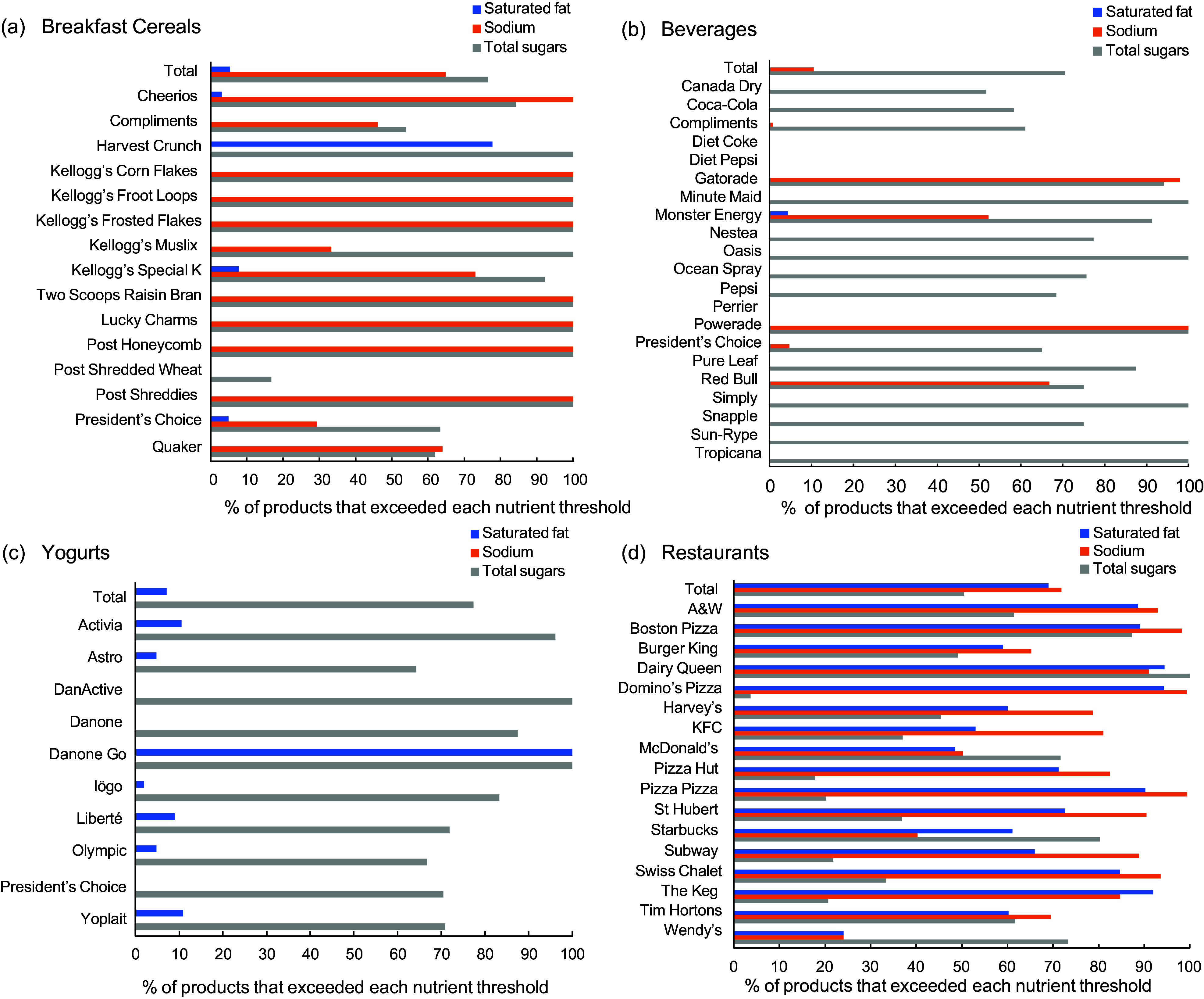
(a–d) The proportion of products offered by the top breakfast cereal, beverage, yogurt and restaurant brand that exceeded Health Canada’s thresholds for saturated fat, Na and/or total sugars for proposed restrictions on food marketing to children. Products exceeding one or more of these thresholds would be restricted from marketing to children*. ^*^Only products containing added Na (e.g. added Na or salt, monosodium glutamate, added cheese or salted nuts, baking soda, etc.) were assessed against the Na threshold, only products containing free sugars (e.g. 100 % and concentrated fruit juice, honey, sugars from artificial flavours or fruit and vegetable purées, dextrose) were assessed against the total sugars threshold and only those with added fat (e.g. vegetable and animal fats and oils, olive oil, butter, margarine, shortening, etc.) were assessed against the saturated fat thresholds. Foods containing no added Na, free sugars or added fat were exempt from the nutrient thresholds.

In total, 73·4 % of packaged foods and beverages exceeded the total sugars threshold, while fewer products exceeded the thresholds for Na (16·2 %) and saturated fat (3·0 %). More than three-quarters of breakfast cereals (76·6 %) exceeded the threshold for total sugars, including 50 % or more of the products offered by all fifteen brands and 100 % of products offered by nine brands (Fig. 4(a)–(d); see online supplementary material, Supplementary Table 3). Similarly, 70·5 % of beverages exceeded the total sugars threshold, which included at least 50 % of the products offered by eighteen brands and 100 % of the products offered by six brands (Fig. 4(a)–(d); see online supplementary material, Supplementary Table 3). Among the yogurts, 77·4 % of products exceeded the total sugars threshold, including 50 % or more of the products offered by all ten brands and 100 % of the products from two brands (DanActive and DanoneGo) (Fig. 4(a)–(d); see online supplementary material, Supplementary Table 3). Lastly, 50·4 % of restaurant menu items exceeded the total sugars threshold, including more than 50 % of items offered by seven brands and 100 % of Dairy Queen menu items (Fig. 4(a)–(d); see online supplementary material, Supplementary Table 3). When evaluated for Na content, 64·9 % of breakfast cereals exceeded the threshold, including at least 50 % of the products offered by eleven brands and 100 % of the products offered by eight brands. Additionally, 10·5 % of beverages exceeded the HC’s M2K threshold for Na, including 50 % or more of the products offered by four brands (Gatorade, Red Bull, Monster Energy and Powerade). No yogurt products exceeded the Na threshold. However, 71·8 % of restaurant menu items exceeded the Na threshold, with fifteen of the seventeen restaurants having 50 % or more products above the threshold. When assessed against the saturated fat threshold, most breakfast cereal (94·6 %) and beverage products (99·8 %) did not exceed it. One exception was Harvest Crunch, where 77·8 % of their breakfast cereals exceeded the saturated fat threshold. Among the yogurts, only 7·1 % of the total products exceeded the saturated fat threshold, including 100 % of DanoneGo products. Lastly, more than two-thirds (69·0 %) of the restaurant menu items in this sample exceeded the saturated fat threshold; for fifteen restaurant brands, this included more than 50 % of their products.

## Discussion

This study found that most products offered by leading breakfast cereal, beverage, yogurt and restaurant brands in Canada would be considered too unhealthy to be marketed to children based on HC’s M2K thresholds for saturated fat, Na and total sugars published in the currently proposed federal regulations. For many brands in this study, this included all or most of their products. In fact, for nearly half of the brands sampled (42 %, *n* 21), 100 % of their products exceeded one or more nutrient thresholds. Additionally, more than half of the products offered by twenty-three brands (46 %) exceeded two nutrient thresholds. These findings reinforce the need not only for regulations to protect Canadian children from marketing of unhealthy foods but also for such restrictions to extend to brand marketing.

Consistent with previous research^(28)^, this study suggests that most packaged foods and beverages in Canada exceed one or more of HC’s proposed nutrient thresholds for restricting unhealthy food marketing to children. Most packaged foods and beverages that would be restricted exceeded the threshold for total sugars (73·4 %), with fewer products exceeding the thresholds for Na (16·2 %) and saturated fat (3·0 %). This is likely, in part, a reflection of the food categories selected for analysis, with breakfast cereals, beverages and yogurts tending to be relatively high in sugars and lower in Na and saturated fat^(27)^. Nonetheless, nearly two-thirds of the breakfast cereals exceeded the Na threshold, including all products offered by eight of the fifteen sampled breakfast cereal brands. This study also provides novel evidence of how restaurant chain menu items would fare against HC’s proposed nutrient thresholds for restricting marketing to children. Overall, compared with packaged foods and beverages, a greater proportion of menu items offered by each restaurant brand exceeded one or more nutrient thresholds, including 93 % of items offered by all restaurant brands. Additionally, all restaurants had menu items that exceeded three nutrient thresholds, ranging as high as 87 % of a restaurant’s menu (Dairy Queen). These findings align with those of previous studies demonstrating the poor nutritional quality of Canadian restaurant menu items^(21,29,30)^ and reinforce the need for nutrition policies to extend to the foodservice sector. HC’s currently proposed food marketing policy would include restaurant foods^(7)^, and our findings indicate that HC’s M2K thresholds have the potential to greatly reduce children’s exposure to marketing of unhealthy restaurant foods, depending on how the regulations are applied (e.g. whether they include brand marketing).

These findings also support the need for policies on restricting food marketing to children to extend to brand marketing. In addition to products and services, the promotion of a related brand is included within the scope of the WHO’s definition of marketing^(5)^. The WHO guidelines also identify branding as a technique used to increase the power of a marketing communication or action^(13)^. Brand marketing fosters brand awareness and preferences and has been shown to negatively affect children’s purchasing behaviours and dietary intakes^(31–35)^. One study found the logos of popular food brands activated regions of children’s brains associated with motivation and cognitive control^(36)^, suggesting that the effectiveness of brand marketing may be related to the neural responses it elicits. In combination with existing evidence, findings of the present study reinforce the potential health consequences of brand marketing, given that most or all products offered by the leading brands in this sample are high in total sugars, Na and/or saturated fat, thereby contributing to high intakes of nutrients of concern. Government regulations to restrict the marketing of unhealthy food brands to children – irrespective of whether an identifiable product is featured or referenced – are therefore warranted.

Despite evidence of the harmful impacts of brand marketing on children and growing calls to include brand marketing in the scope of policies, current regulations and voluntary initiatives typically permit it since a specific product is not promoted^(5,6)^. Excluding brand marketing from a policy may result in increased brand advertising and sponsorship^(5,37)^ by brands with large proportions of unhealthy products (such as those in this sample). The WHO recommends countries consider restricting brands that are closely linked with less healthy products or assess the healthfulness of a brand’s best-selling products to determine whether the brand should be permitted to be marketed^(14)^. This study demonstrates that determining the proportion of a brand’s total products that is too unhealthy to be marketed is a potentially feasible approach. For example, restricting brands with 50 % or more of their products considered ‘unhealthy’ (according to HC’s nutrient profile model) would prevent virtually all brands (92·0 %, *n* 46) in this sample from marketing to children. The only exceptions would be brands only offering products low in all three nutrients of concern (e.g. diet soft drink or carbonated water brands, whole-grain unsweetened cereal brands). Given that historically, food marketing policies have focused on restricting specific foods, policymakers may be challenged to develop restrictions on brand marketing where no food is present. Nonetheless, this study demonstrates the potential for brand product healthfulness thresholds (e.g. restricting marketing of brands where ≥50 % of products exceed nutrient thresholds or fail a nutrient profile model) could be used as an indicator of brand healthfulness in marketing policies and regulations. Other researchers have proposed using food sales data to determine whether a brand or company product portfolio is considered sufficiently healthy to be marketed to children. For example, Bandy et al. (2023) suggest mandating that the minority of a company’s sales be from unhealthy foods (e.g. <50 %, <25 % or <10 %) or a reduction in the proportion of sales from unhealthy products over time^(38)^. Similarly, The George Institute of Global Health (Australia) recommends that classifying brands according to healthfulness be based on sales rather than product range, with continued re-assessment of sales and brand classification on a regular basis^(39)^. There is, however, a need for further research to evaluate the healthfulness of a broader proportion of food categories and brands within companies’ product portfolios. The present study was limited to breakfast cereals, beverages and yogurt – some of the top food categories marketed to children^(27)^; however, certain brands in this sample also offer products in other food categories, some of which may be considered ‘healthier’ according to HC’s nutrient profile model.

The policy currently proposed by the Canadian government to restrict unhealthy food marketing to children omits brand marketing^(7)^. This means that provided no identifiable product is included in a marketing communication, most of the brands in this sample would be permitted to be marketed, irrespective of whether they exceed HC’s nutrient thresholds. For example, brands like McDonald’s and Coca-Cola – two of the best-selling and most recognisable brands globally^(40,41)^ – would still be allowed to market their brands using their famous logos (e.g. Coca-Cola’s red and white logo), brand slogans (e.g. McDonald’s ‘I’m Lovin’ It’) and spokes characters (e.g. McDonald’s Ronald McDonald) to appeal to children, or by sponsoring events where children are present (e.g. children’s sports teams and major sporting events). This is concerning given that a large proportion of McDonald’s (88·4 %) and Coca-Cola (58·3 %) products are high in nutrients of concern and exceed HC’s proposed thresholds, and that these brands are inextricably linked to unhealthy products (hamburgers and sweetened cola, respectively)^(42,43)^. Because these brands are synonymous with unhealthy foods, even advertising healthier products offered by these brands (e.g. McDonald’s salads or Happy Meals with apple slices) may have detrimental impacts on food choices and diet quality^(44)^. In addition, food companies such as these are increasingly employing brand marketing strategies that blur the line between entertainment and advertising, particularly in digital media, making it more difficult for children to identify marketing instances (e.g. advergames, branded content on social media and influencer marketing, sponsorship of sporting events)^(5,45)^. Given our findings, pre-existing evidence and WHO recommendations, HC and other governments should incorporate brand marketing within the scope of their policy to maximise its potential to limit children’s exposure to unhealthy food marketing.

This study indicates how Canada’s leading packaged food, beverage and restaurant brands would fare against HC’s nutrient profile model for their proposed policy to restrict unhealthy food marketing to children. While previous research has evaluated the nutritional quality of packaged food and beverages against HC’s nutrient profile model^(28)^, this study was novel in its application of the thresholds to restaurant foods, which are included in the current policy proposal. Our sample also consisted of products offered by the top-selling breakfast cereal, beverage and yogurt brands in Canada, thereby representing the leading products most consumed by Canadians. Additionally, since many of the sampled brands are multinational, results of this study may be somewhat generalisable to other markets. While a growing number of studies have examined the nutritional quality of packaged foods and beverages at the company/manufacturer level^(46–48)^, less is known about the healthfulness of individual brands offered by food companies. These findings may be used as evidence to push for brand marketing to be included in food marketing regulations proposed or implemented in other countries. Nonetheless, this study is not without limitations. First, data collection was limited to products available on grocery retailer and restaurant brand websites at a single point in time and may therefore not capture all products offered by the sampled brands in Canada. It is also possible that some products were reformulated, discontinued or newly introduced since data were collected in 2020. Furthermore, due to the lack of nutrition labelling regulations for online grocery retail, the presentation of nutrition information on grocery retailer and restaurant brand websites is not mandatory or standardised, resulting in limited availability and consistency of NFt data and ingredients lists on some websites. There was, however, very little missing data for the products offered by the brands in this sample, with only thirty-three menu items excluded due to missing nutritional information. Lastly, individual products in this sample were not sales-weighted due to the fact the availability of product-level sales data in Canada is limited and very expensive to acquire.

Overall, this study found that most products offered by the top breakfast cereal, beverage, yogurt and restaurant brands in Canada are high in total sugars, Na and/or saturated fat and would not be permitted to be marketed to children under HC’s proposed regulations. However, given that brand marketing is not currently included within the scope of the proposed policy, brands with predominantly unhealthy products would still be able to indirectly market to children through communications that do not feature or reference specific products, using their well-known and highly recognisable logos, slogans, spokes characters and other brand marketing techniques, or via sponsorship. Our findings reinforce the need for Canada and other countries to consider including brand marketing in regulations aimed at reducing children’s exposure to unhealthy food marketing. Future studies on the impact of brand marketing and potential unintended consequences of excluding it from regulations are warranted to help close this policy loophole.

## Supporting information

Vergeer et al. supplementary material Vergeer et al. supplementary material

## Supplementary material

For supplementary material accompanying this paper visit https://doi.org/10.1017/S1368980024002659
