# Supplementary material for: The healthfulness of major food brands according to Health Canada’s nutrient profile model for proposed restrictions on food marketing to children
Source: Public Health Nutr. 2025 Jan 7;28(1):e17. doi: 10.1017/S1368980024002659 (PMC11822582; doi:10.1017/S1368980024002659)
Supplement: Vergeer et al. supplementary material [file S1368980024002659sup001.pdf]

SUPPLEMENTARY MATERIAL

TITLE: The healthfulness of major food brands according to Health Canada's nutrient profile model for proposed restrictions on food marketing to children

AUTHORS: Laura Vergeer[1], Christine Mulligan[1], Hayun (Sarah) Jeong[1], Ayesha Khan[1], Mary R. L'Abbé[1]

AFFILIATIONS: [1]Department of Nutritional Sciences, Temerty Faculty of Medicine, University of Toronto, 1 King's College Circle, Toronto, ON, Canada M5S 1A8

Supplementary Table 1. The proportion and number of products offered the top breakfast cereal, beverage, yogurt and restaurant brands that would be permitted and restricted from marketing to children (M2K) based on the nutrient thresholds proposed by Health Canada (HC).*

Brand	Total number of products†	Permitted to be M2K						Restricted from M2K	
		Exempt from evaluation‡		Not exempt from evaluation and does not exceed HC's M2K thresholds		Total products permitted for M2K		Not exempt from evaluation and exceeds HC's M2K thresholds	
	n	%	n	%	n	%	n	%	n
Packaged food/beverages (total)*	1385	16.3	226	8.9	123	25.2	349	74.8	1036
Breakfast cereals									
**Total**	222	11.7	26	5.4	12	17.1	38	82.9	184
Cheerios	32	0.0	0	0.0	0	0.0	0	100.0	32
Compliments	13	0.0	0	23.1	3	23.1	3	76.9	10
Harvest Crunch	9	0.0	0	0.0	0	0.0	0	100.0	9
Kellogg's Corn Flakes	4	0.0	0	0.0	0	0.0	0	100.0	4
Kellogg's Froot Loops	6	0.0	0	0.0	0	0.0	0	100.0	6
Kellogg's Frosted Flakes	6	0.0	0	0.0	0	0.0	0	100.0	6
Kellogg's Muslix	3	0.0	0	0.0	0	0.0	0	100.0	3
Kellogg's Special K	26	7.7	2	0.0	0	7.7	2	92.3	24
Kellogg's Two Scoops Raisin Bran	4	0.0	0	0.0	0	0.0	0	100.0	4
Lucky Charms	11	0.0	0	0.0	0	0.0	0	100.0	11
Post Honeycomb	4	0.0	0	0.0	0	0.0	0	100.0	4
Post Shredded Wheat	6	83.3	5	0.0	0	83.3	5	16.7	1

Brand	Total number of products[†]	Permitted to be M2K						Restricted from M2K	
		Exempt from evaluation[‡]		Not exempt from evaluation and does not exceed HC's M2K thresholds		Total products permitted for M2K		Not exempt from evaluation and exceeds HC's M2K thresholds	
	n	%	n	%	n	%	n	%	n
Post Shreddies	7	0.0	0	0.0	0	0.0	0	100.0	7
President's Choice	41	12.2	5	14.6	6	26.8	11	73.2	30
Quaker	50	28.0	14	6.0	3	34.0	17	66.0	33
Beverages									
**Total**	769	16.1	124	12.7	98	28.8	222	71.1	547
Canada Dry	29	37.9	11	10.3	3	48.2	14	51.7	15
Coca-Cola	24	41.7	10	0.0	0	41.7	10	58.3	14
Compliments	118	3.4	4	34.7	41	38.1	45	61.9	73
Diet Coke	27	29.6	8	70.4	19	100.0	27	0.0	0
Diet Pepsi	14	21.4	3	78.6	11	100.0	14	0.0	0
Gatorade	51	0.0	0	2.0	1	2.0	1	98.0	50
Minute Maid	61	0.0	0	0.0	0	0.0	0	100.0	61
Monster Energy	23	0.0	0	8.7	2	8.7	2	91.3	21
Nestea	22	22.7	5	0.0	0	22.7	5	77.3	17
Oasis	67	0.0	0	0.0	0	0.0	0	100.0	67
Ocean Spray	45	0.0	0	24.4	11	24.4	11	75.6	34
Pepsi	19	31.6	6	0.0	0	31.6	6	68.4	13
Perrier	38	97.4	37	2.6	1	100.0	38	0.0	0
Powerade	4	0.0	0	0.0	0	0.0	0	100.0	4
President's Choice	129	29.5	38	4.7	6	34.2	44	65.9	85
Pure Leaf	16	12.5	2	0.0	0	12.5	2	87.5	14
Red Bull	12	0.0	0	16.7	2	16.7	2	83.3	10
Simply	19	0.0	0	0.0	0	0.0	0	100.0	19
Snapple	4	0.0	0	25.0	1	25.0	1	75.0	3
Sun-Rype	12	0.0	0	0.0	0	0.0	0	100.0	12

Brand	Total number of products†	Permitted to be M2K						Restricted from M2K	
		Exempt from evaluation‡		Not exempt from evaluation and does not exceed HC's M2K thresholds		Total products permitted for M2K		Not exempt from evaluation and exceeds HC's M2K thresholds	
	n	%	n	%	n	%	n	%	n
Tropicana	35	0.0	0	0.0	0	0.0	0	100.0	35
Yogurts									
**Total**	394	19.3	76	3.3	13	22.6	89	77.4	305
Activia	76	3.9	3	0.0	0	3.9	3	96.1	73
Astro	42	31.0	13	4.8	2	35.8	15	64.3	27
DanActive	3	0.0	0	0.0	0	0.0	0	100.0	3
Danone	8	12.5	1	0.0	0	12.5	1	87.5	7
Danone Go	2	0.0	0	0.0	0	0.0	0	100.0	2
Iögo	54	14.8	8	1.9	1	16.7	9	83.3	45
Liberté	89	28.1	25	0.0	0	28.1	25	71.9	64
Olympic	21	33.3	7	0.0	0	33.3	7	66.7	14
President's Choice	44	29.5	13	0.0	0	29.5	13	70.5	31
Yoplait	55	10.9	6	18.2	10	29.1	16	70.9	39
**Restaurants**									
**Total**	3153	5.7	181	1.3	40	7.0	221	93.0	2932
A&W	114	3.5	4	0.0	0	3.5	4	96.5	110
Boston Pizza	55	1.8	1	0.0	0	1.8	1	98.2	54
Burger King	161	11.8	19	0.0	0	11.8	19	88.2	142
Dairy Queen	178	0.0	0	0.0	0	0.0	0	100.0	178
Domino's Pizza	140	0.0	0	0.0	0	0.0	0	100.0	140
Harvey's	75	0.0	0	2.7	2	2.7	2	97.3	73
KFC	100	2.0	2	1.0	1	3.0	3	97.0	97
McDonald's	225	10.7	24	0.9	2	11.6	26	88.4	199
Pizza Hut	486	4.1	20	1.6	8	5.7	28	94.2	458
Pizza Pizza	163	0.0	0	0.6	1	0.6	1	99.4	162
St Hubert	95	0.0	0	1.1	1	1.1	1	98.9	94

Brand	Total number of products†	Permitted to be M2K						Restricted from M2K	
		Exempt from evaluation‡		Not exempt from evaluation and does not exceed HC's M2K thresholds		Total products permitted for M2K		Not exempt from evaluation and exceeds HC's M2K thresholds	
	n	%	n	%	n	%	n	%	n
Starbucks	640	11.9	76	2.2	14	14.1	90	85.9	550
Subway	188	0.5	1	0.0	0	0.5	1	99.5	187
Swiss Chalet	78	0.0	0	0.0	0	0.0	0	100.0	78
The Keg	111	0.9	1	1.8	2	2.7	3	97.3	108
Tim Hortons	269	7.8	21	1.1	3	8.9	24	91.1	245
Wendy's	75	16.0	12	8.0	6	24.0	18	76.0	57

*The total number and percentage of products in the breakfast cereals, beverages and yogurts categories (combined) from the top food brands with ≥1% market share. For packaged foods, food categories were derived by combining the Table of Reference Amounts for Food minor categories as follows: breakfast cereals (C1, C2, C3, C4); beverages (B1 and J11); and yogurts (D12 and D15). For restaurant menu items, all items in the Menu-FLIP 2020 database offered by the sampled brands with ≥1% market share were analyzed (unless nutrition information was missing). †The total number of products in the FLIP 2020 database (breakfast cereals, beverages and yogurts) or menu items in the Menu-FLIP 2020 database (restaurants) offered under each brand. ‡Products or menu items without free sugars, added sodium or added fat were exempted from all of HC's M2K thresholds.

Supplementary Table 2. The proportion and number of products offered by each breakfast cereal, beverage, yogurt and restaurant brand that exceeded 0, 1, 2 or 3 of Health Canada's nutrient thresholds for proposed restrictions on food marketing to children. Products exceeding one or more nutrient thresholds would be restricted from marketing to children.

**Brand**	**Total number of products**[†]	**Number of thresholds exceeded**[‡]							
		**0**[§]		**1**		**2**		**3**	
	**n**	**%**	**n**	**%**	**n**	**%**	**n**	**%**	**n**
Packaged food/beverages (total)[*]	1385	25.2	349	57.2	792	17.4	241	0.2	3
Breakfast cereals									
**Total**	222	17.1	38	19.8	44	62.2	138	0.9	2
Cheerios	32	0.0	0	15.6	6	81.3	26	3.1	1
Compliments	13	23.1	3	53.8	7	23.1	3	0.0	0
Harvest Crunch	9	0.0	0	22.2	2	77.8	7	0.0	0
Kellogg's Corn Flakes	4	0.0	0	0.0	0	100.0	4	0.0	0
Kellogg's Froot Loops	6	0.0	0	0.0	0	100.0	6	0.0	0
Kellogg's Frosted Flakes	6	0.0	0	0.0	0	100.0	6	0.0	0
Kellogg's Muslix	3	0.0	0	66.7	2	33.3	1	0.0	0
Kellogg's Special K	26	7.7	2	15.4	4	73.1	19	3.8	1
Kellogg's Two Scoops Raisin Bran	4	0.0	0	0.0	0	100.0	4	0.0	0
Lucky Charms	11	0.0	0	0.0	0	100.0	11	0.0	0
Post Honeycomb	4	0.0	0	0.0	0	100.0	4	0.0	0
Post Shredded Wheat	6	83.3	5	16.7	1	0.0	0	0.0	0
Post Shreddies	7	0.0	0	0.0	0	100.0	7	0.0	0
President's Choice	41	26.8	11	48.8	20	24.4	10	0.0	0
Quaker	50	34.0	17	6.0	3	60.0	30	0.0	0
Beverages									
**Total**	769	28.9	222	61.2	471	9.8	75	0.1	1
Canada Dry	29	48.3	14	51.7	15	0.0	0	0.0	0
Coca-Cola	24	41.7	10	58.3	14	0.0	0	0.0	0
Compliments	118	38.1	45	61.9	73	0.0	0	0.0	0
Diet Coke	27	100.0	27	0.0	0	0.0	0	0.0	0
Diet Pepsi	14	100.0	14	0.0	0	0.0	0	0.0	0

**Brand**	**Total number of products**†	**Number of thresholds exceeded**‡							
		**0**§		**1**		**2**		**3**	
	**n**	**%**	**n**	**%**	**n**	**%**	**n**	**%**	**n**
Gatorade	51	2.0	1	3.9	2	94.1	48	0.0	0
Minute Maid	61	0.0	0	100.0	61	0.0	0	0.0	0
Monster Energy	23	8.7	2	39.1	9	47.8	11	4.3	1
Nestea	22	22.7	5	77.3	17	0.0	0	0.0	0
Oasis	67	0.0	0	100.0	67	0.0	0	0.0	0
Ocean Spray	45	24.4	11	75.6	34	0.0	0	0.0	0
Pepsi	19	31.6	6	68.4	13	0.0	0	0.0	0
Perrier	38	100.0	38	0.0	0	0.0	0	0.0	0
Powerade	4	0.0	0	0.0	0	100.0	4	0.0	0
President's Choice	129	34.1	44	62.0	80	3.9	5	0.0	0
Pure Leaf	16	12.5	2	87.5	14	0.0	0	0.0	0
Red Bull	12	16.7	2	25.0	3	58.3	7	0.0	0
Simply	19	0.0	0	100.0	19	0.0	0	0.0	0
Snapple	4	25.0	1	75.0	3	0.0	0	0.0	0
Sun-Rype	12	0.0	0	100.0	12	0.0	0	0.0	0
Tropicana	35	0.0	0	100.0	35	0.0	0	0.0	0
Yogurts									
**Total**	394	22.6	89	70.3	277	7.1	28	0.0	0
Activia	76	3.9	3	85.5	65	10.5	8	0.0	0
Astro	42	35.7	15	59.5	25	4.8	2	0.0	0
DanActive	3	0.0	0	100.0	3	0.0	0	0.0	0
Danone	8	12.5	1	87.5	7	0.0	0	0.0	0
Danone Go	2	0.0	0	0.0	0	100.0	2	0.0	0
Iögo	54	16.7	9	81.5	44	1.9	1	0.0	0
Liberté	89	28.1	25	62.9	56	9.0	8	0.0	0
Olympic	21	33.3	7	61.9	13	4.8	1	0.0	0
President's Choice	44	29.5	13	70.5	31	0.0	0	0.0	0
Yoplait	55	29.1	16	60.0	33	10.9	6	0.0	0
Restaurants									
**Total**	3153	6.3	198	19.7	620	48.4	1527	25.6	808

Brand	Total number of products†	Number of thresholds exceeded‡							
		0§		1		2		3	
	n	%	n	%	n	%	n	%	n
A&W	114	0.9	1	9.6	11	32.5	37	57.0	65
Boston Pizza	55	0.0	0	0.0	0	21.8	12	78.2	43
Burger King	161	9.9	16	24.8	40	45.3	73	19.9	32
Dairy Queen	178	0.0	0	1.7	3	11.2	20	87.1	155
Domino's Pizza	140	0.0	0	5.0	7	92.9	130	2.1	3
Harvey's	75	2.7	2	34.7	26	38.7	29	24.0	18
KFC	100	3.0	3	36.0	36	48.0	48	13.0	13
McDonald's	225	10.7	24	32.4	73	31.1	70	25.8	58
Pizza Hut	486	5.6	27	19.3	94	72.6	353	2.5	12
Pizza Pizza	163	0.6	1	4.3	7	79.8	130	15.3	25
St Hubert	95	1.1	1	15.8	15	64.2	61	18.9	18
Starbucks	640	12.7	81	23.0	147	29.7	190	34.7	222
Subway	188	0.5	1	26.1	49	68.1	128	5.3	10
Swiss Chalet	78	0.0	0	14.1	11	59.0	46	26.9	21
The Keg	111	1.8	2	4.5	5	80.2	89	13.5	15
Tim Hortons	269	8.6	23	21.9	59	38.3	103	31.2	84
Wendy's	75	21.3	16	49.3	37	10.7	8	18.7	14

*The total number and percentage of products in the breakfast cereals, beverage and yogurts categories (combined). For packaged foods, food categories were derived by combining the Table of Reference Amounts for Food minor categories as follows: breakfast cereals (C1, C2, C3, C4); beverage (B1 and J11); and yogurts (D12 and D15). For restaurant menu items, all items in the Menu-FLIP 2020 database offered by the sampled brands were analyzed (unless nutrition information was missing). †The total number of products in the FLIP 2020 database (breakfast cereals, beverages and yogurts) or menu items in the Menu-FLIP 2020 database (restaurants) offered under each brand. ‡The number and percentage of products that exceeded 0, 1, 2 or 3 of Health Canada's thresholds for sodium, saturated fat and/or total sugars for proposed restrictions on food marketing to children. Products or menu items without free sugars, added sodium or added fat were exempted from all of Health Canada's nutrient thresholds. §Includes products that contained added sodium, added fat and/or free sugars but did not exceed any nutrient thresholds, as well as products that did not contain added sodium, added fat or free sugars and were therefore exempt from evaluation against the thresholds.

Supplementary Table 3. The proportion and number of products offered by the top breakfast cereal, beverage, yogurt and restaurant brand that exceeded Health Canada's thresholds for saturated fat, sodium and/or total sugars for proposed restrictions on food marketing to children. Products exceeding one or more of these thresholds would be restricted from marketing to children.

		Saturated fat						Sodium						Total sugars					
**Brand**	**Total number of products**[†]	**Exempt from evaluation**[‡]		**Not exempt, does not exceed threshold**		**Not exempt, exceeds threshold**		**Exempt from evaluation**[‡]		**Not exempt, does not exceed threshold**		**Not exempt, exceeds threshold**		**Exempt from evaluation**[‡]		**Not exempt, does not exceed threshold**		**Exceeds total sugars threshold**	
	**n**	**%**	**n**	**%**	**n**	**%**	**n**	**%**	**n**	**%**	**n**	**%**	**n**	**%**	**n**	**%**	**n**	**%**	**n**
Packaged food/beverages (total)[*]	1385	11.6	161	85.4	1183	3.0	41	0.1	2	83.6	1158	16.2	225	2.2	31	24.3	337	73.4	1017
Breakfast cereals																			
**Total**	222	0.5	1	94.1	209	5.4	12	0.9	2	34.2	76	64.9	144	1.4	3	22.1	49	76.6	170
Cheerios	32	0.0	0	96.9	31	3.1	1	0.0	0	0.0	0	100.0	32	0.0	0	15.6	5	84.4	27
Compliments	13	0.0	0	100.0	13	0.0	0	0.0	0	53.8	7	46.2	6	0.0	0	46.2	6	53.8	7
Harvest Crunch	9	0.0	0	22.2	2	77.8	7	0.0	0	100.0	9	0.0	0	0.0	0	0.0	0	100.0	9
Kellogg's Corn Flakes	4	0.0	0	100.0	4	0.0	0	0.0	0	0.0	0	100.0	4	0.0	0	0.0	0	100.0	4
Kellogg's Froot Loops	6	0.0	0	100.0	6	0.0	0	0.0	0	0.0	0	100.0	6	0.0	0	0.0	0	100.0	6
Kellogg's Frosted Flakes	6	0.0	0	100.0	6	0.0	0	0.0	0	0.0	0	100.0	6	0.0	0	0.0	0	100.0	6
Kellogg's Muslix	3	0.0	0	100.0	3	0.0	0	0.0	0	66.7	2	33.3	1	0.0	0	0.0	0	100.0	3
Kellogg's Special K	26	0.0	0	92.3	24	7.7	2	7.7	2	19.2	5	73.1	19	7.7	2	0.0	0	92.3	24

Brand	Total number of products†	Saturated fat: Exempt from evaluation‡		Saturated fat: Not exempt, does not exceed threshold		Saturated fat: Not exempt, exceeds threshold		Sodium: Exempt from evaluation‡		Sodium: Not exempt, does not exceed threshold		Sodium: Not exempt, exceeds threshold		Total sugars: Exempt from evaluation‡		Total sugars: Not exempt, does not exceed threshold		Total sugars: Exceeds total sugars threshold	
	n	%	n	%	n	%	n	%	n	%	n	%	n	%	n	%	n	%	n
Kellogg's Two Scoops Raisin Bran	4	0.0	0	100.0	4	0.0	0	0.0	0	0.0	0	100.0	4	0.0	0	0.0	0	100.0	4
Lucky Charms	11	0.0	0	100.0	11	0.0	0	0.0	0	0.0	0	100.0	11	0.0	0	0.0	0	100.0	11
Post Honeycomb	4	0.0	0	100.0	4	0.0	0	0.0	0	0.0	0	100.0	4	0.0	0	0.0	0	100.0	4
Post Shredded Wheat	6	0.0	0	100.0	6	0.0	0	0.0	0	100.0	6	0.0	0	0.0	0	83.3	5	16.7	1
Post Shreddies	7	0.0	0	100.0	7	0.0	0	0.0	0	0.0	0	100.0	7	0.0	0	0.0	0	100.0	7
President's Choice	41	0.0	0	95.1	39	4.9	2	0.0	0	70.7	29	29.3	12	2.4	1	34.1	14	63.4	26
Quaker	50	2.0	1	98.0	49	0.0	0	0.0	0	36.0	18	64.0	32	0.0	0	38.0	19	62.0	31
Beverages																			
**Total**	769	0.1	1	99.7	767	0.1	1	0.0	0	89.5	688	10.5	81	0.3	2	29.3	225	70.5	542
Canada Dry	29	0.0	0	100.0	29	0.0	0	0.0	0	100.0	29	0.0	0	0.0	0	48.3	14	51.7	15
Coca-Cola	24	0.0	0	100.0	24	0.0	0	0.0	0	100.0	24	0.0	0	0.0	0	41.7	10	58.3	14
Compliments	118	0.0	0	100.0	118	0.0	0	0.0	0	99.2	117	0.8	1	0.0	0	39.0	46	61.0	72
Diet Coke	27	0.0	0	100.0	27	0.0	0	0.0	0	100.0	27	0.0	0	0.0	0	100.0	27	0.0	0
Diet Pepsi	14	0.0	0	100.0	14	0.0	0	0.0	0	100.0	14	0.0	0	0.0	0	100.0	14	0.0	0

		Saturated fat						Sodium						Total sugars					
**Brand**	**Total number of products[†]**	**Exempt from evaluation[‡]**		**Not exempt, does not exceed threshold**		**Not exempt, exceeds threshold**		**Exempt from evaluation[‡]**		**Not exempt, does not exceed threshold**		**Not exempt, exceeds threshold**		**Exempt from evaluation[‡]**		**Not exempt, does not exceed threshold**		**Exceeds total sugars threshold**	
	**n**	**%**	**n**	**%**	**n**	**%**	**n**	**%**	**n**	**%**	**n**	**%**	**n**	**%**	**n**	**%**	**n**	**%**	**n**
Gatorade	51	0.0	0	100.0	51	0.0	0	0.0	0	2.0	1	98.0	50	0.0	0	5.9	3	94.1	48
Minute Maid	61	0.0	0	100.0	61	0.0	0	0.0	0	100.0	61	0.0	0	0.0	0	100.0	61	100.0	61
Monster Energy	23	4.3	1	91.3	21	4.3	1	0.0	0	47.8	11	52.2	12	0.0	0	8.7	2	91.3	21
Nestea	22	0.0	0	100.0	22	0.0	0	0.0	0	100.0	22	0.0	0	0.0	0	22.7	5	77.3	17
Oasis	67	0.0	0	100.0	67	0.0	0	0.0	0	100.0	67	0.0	0	0.0	0	0.0	0	100.0	67
Ocean Spray	45	0.0	0	100.0	45	0.0	0	0.0	0	100.0	45	0.0	0	0.0	0	11	24.4	75.6	34
Pepsi	19	0.0	0	100.0	19	0.0	0	0.0	0	100.0	19	0.0	0	0.0	0	31.6	6	68.4	13
Perrier	38	0.0	0	100.0	38	0.0	0	0.0	0	100.0	38	0.0	0	0.0	0	100.0	38	0.0	0
Powerade	4	0.0	0	100.0	4	0.0	0	0.0	0	0.0	0	100.0	4	0.0	0	0.0	0	100.0	4
President's Choice	129	0.0	0	100.0	129	0.0	0	0.0	0	95.3	123	4.7	6	1.6	2	33.3	43	65.1	84
Pure Leaf	16	0.0	0	100.0	16	0.0	0	0.0	0	100.0	16	0.0	0	0.0	0	12.5	2	87.5	14
Red Bull	12	0.0	0	100.0	12	0.0	0	0.0	0	33.3	4	66.7	8	0.0	0	25.0	3	75.0	9
Simply	19	0.0	0	100.0	19	0.0	0	0.0	0	100.0	19	0.0	0	0.0	0	0.0	0	100.0	19
Snapple	4	0.0	0	100.0	4	0.0	0	0.0	0	100.0	4	0.0	0	0.0	0	25.0	1	75.0	3

		Saturated fat						Sodium						Total sugars					
**Brand**	**Total number of products†**	**Exempt from evaluation‡**		**Not exempt, does not exceed threshold**		**Not exempt, exceeds threshold**		**Exempt from evaluation‡**		**Not exempt, does not exceed threshold**		**Not exempt, exceeds threshold**		**Exempt from evaluation‡**		**Not exempt, does not exceed threshold**		**Exceeds total sugars threshold**	
	**n**	**%**	**n**	**%**	**n**	**%**	**n**	**%**	**n**	**%**	**n**	**%**	**n**	**%**	**n**	**%**	**n**	**%**	**n**
Sun-Rype	12	0.0	0	100.0	12	0.0	0	0.0	0	100.0	12	0.0	0	0.0	0	0.0	0	100.0	12
Tropicana	35	0.0	0	100.0	35	0.0	0	0.0	0	100.0	35	0.0	0	0.0	0	0.0	0	100.0	35
Yogurts																			
**Total**	394	40.4	159	52.5	207	7.1	28	0.0	0	100.0	394	0.0	0	6.6	26	16.0	63	77.4	305
Activia	76	43.4	33	46.1	35	10.5	8	0.0	0	100.0	76	0.0	0	2.6	2	1.3	1	96.1	73
Astro	42	71.4	30	23.8	10	4.8	2	0.0	0	100.0	42	0.0	0	7.1	3	28.6	12	64.3	27
DanActive	3	100.0	3	0.0	0	0.0	0	0.0	0	100.0	3	0.0	0	0.0	0	0.0	0	100.0	3
Danone	8	12.5	1	87.5	7	0.0	0	0.0	0	100.0	8	0.0	0	0.0	0	12.5	1	87.5	7
Danone Go	2	0.0	0	0.0	0	100.0	2	0.0	0	100.0	2	0.0	0	0.0	0	0.0	0	100.0	2
Iögo	54	24.1	13	74.1	40	1.9	1	0.0	0	100.0	54	0.0	0	0.0	0	16.7	9	83.3	45
Liberté	89	36.0	32	55.1	49	9.0	8	0.0	0	100.0	89	0.0	0	18.0	16	10.1	9	71.9	64
Olympic	21	85.7	18	9.5	2	4.8	1	0.0	0	100.0	21	0.0	0	14.3	3	19.0	4	66.7	14
President's Choice	44	22.7	10	77.3	34	0.0	0	0.0	0	100.0	44	0.0	0	4.5	2	25.0	11	70.5	31
Yoplait	55	34.5	19	54.5	30	10.9	6	0.0	0	100.0	55	0.0	0	0.0	0	29.1	16	70.9	39
Restaurants																			

		Saturated fat						Sodium						Total sugars					
**Brand**	**Total number of products†**	**Exempt from evaluation‡**		**Not exempt, does not exceed threshold**		**Not exempt, exceeds threshold**		**Exempt from evaluation‡**		**Not exempt, does not exceed threshold**		**Not exempt, exceeds threshold**		**Exempt from evaluation‡**		**Not exempt, does not exceed threshold**		**Exceeds total sugars threshold**	
	**n**	**%**	**n**	**%**	**n**	**%**	**n**	**%**	**n**	**%**	**n**	**%**	**n**	**%**	**n**	**%**	**n**	**%**	**n**
**Total**	3153	5.7	181	25.2	795	69.0	2177	5.7	181	22.4	707	71.8	2265	5.7	181	43.8	1382	50.4	1590
A&W	114	3.5	4	7.9	9	88.6	101	3.5	4	3.5	4	93.0	106	3.5	4	35.1	40	61.4	70
Boston Pizza	55	1.8	1	9.1	5	89.1	49	1.8	1	0.0	0	98.2	54	1.8	1	10.9	6	87.3	48
Burger King	161	11.8	19	29.2	47	59.0	95	11.8	19	23.0	37	65.2	105	11.8	19	39.1	63	49.1	79
Dairy Queen	178	0.0	0	5.6	10	94.4	168	0.0	0	9.0	16	91.0	162	0.0	0	0.0	0	100.0	178
Domino's Pizza	140	0.0	0	5.7	8	94.3	132	0.0	0	0.7	1	99.3	139	0.0	0	96.4	135	3.6	5
Harvey's	75	0.0	0	40.0	30	60.0	45	0.0	0	21.3	16	78.7	59	0.0	0	54.7	41	45.3	34
KFC	100	2.0	2	45.0	45	53.0	53	2.0	2	17.0	17	81.0	81	2.0	2	61.0	61	37.0	37
McDonald's	225	10.7	24	40.9	92	48.4	109	10.7	24	39.1	88	50.2	113	10.7	24	17.8	40	71.6	161
Pizza Hut	486	4.1	20	24.7	120	71.2	346	4.1	20	13.4	65	82.5	401	4.1	20	78.2	380	17.7	86
Pizza Pizza	163	0.0	0	9.8	16	90.2	147	0.0	0	0.6	1	99.4	162	0.0	0	79.8	130	20.2	33
St Hubert	95	0.0	0	27.4	26	72.6	69	0.0	0	9.5	9	90.5	86	0.0	0	63.2	60	36.8	35
Starbucks	640	11.9	76	27.0	173	61.1	391	11.9	76	47.8	306	40.3	258	11.9	76	8.0	51	80.2	513
Subway	188	0.5	1	33.5	63	66.0	124	0.5	1	10.6	20	88.8	167	0.5	1	77.7	146	21.8	41
Swiss Chalet	78	0.0	0	15.4	12	84.6	66	0.0	0	6.4	5	93.6	73	0.0	0	66.7	52	33.3	26
The Keg	111	0.9	1	7.2	8	91.9	102	0.9	1	14.4	16	84.7	94	0.9	1	78.4	87	20.7	23
Tim Hortons	269	7.8	21	32.0	86	60.2	162	7.8	21	22.7	61	69.5	187	7.8	21	30.5	82	61.7	166
Wendy's	75	16.0	12	60.0	45	24.0	18	16.0	12	60.0	45	24.0	18	16.0	12	10.7	8	73.3	55

*The total number and percentage of products in the breakfast cereals, beverage and yogurts categories (combined) from the top brands with ≥1% market share. For packaged foods, food categories were derived by combining the Table of Reference Amounts for Food minor categories as follows: breakfast cereals (C1, C2,

C3, C4); beverages (B1 and J11); and yogurts (D12 and D15). For restaurant menu items, all items in the Menu-FLIP 2020 database offered by the sampled brands were analyzed (unless nutrition information was missing). †The total number of products in the FLIP 2020 database (breakfast cereals, beverages and yogurts) or menu items in the Menu-FLIP 2020 database (restaurants) offered under each brand. ‡Only products containing added sodium (e.g., added sodium or salt, monosodium glutamate, added cheese or salted nuts, baking soda, etc.) were assessed against the sodium threshold, only products containing free sugars (e.g., 100% and concentrated fruit juice, honey, sugars from artificial flavours or fruit and vegetable purées, dextrose) were assessed against the total sugars threshold, and only those with added fat (e.g., vegetable and animal fats and oils, olive oil, butter, margarine, shortening, etc.) were assessed against the saturated fat thresholds. Foods containing no added sodium, free sugars or added fat were exempt from the nutrient thresholds.
